# The Tragical Historie of Our Late Brother, Robert, Earl of Essex

**Published:** 1895-08

**Authors:** 


### BOOK NOTICES.

The Tragical Historie of our Late Brother, Robert, Earl of Essex. By the Author of Hamlet, Richard III, Othello, As You Like It, etc., and of the newly discovered tragedy, Mary Queen of Scots. Deciphered from the works of Sir Francis Bacon by Orville W. Owen, M. D. Detroit, Michigan: Howard Publishing Company. London: Gay & Bird, 5 Chandos Street. Paper cover, price, 50 cents.

This volume is still another one of the wonderful discoveries made by Dr. Orville Owen in the text of the plays attributed to Shakespeare by means of the wonderful cipher-key.

In this story, which is constructed in the form of a play, it is revealed that the Earl of Essex was the son of Queen Elizabeth, and therefore own brother of Francis Bacon. Essex was of a frank and generous nature, hot-headed and rash.

Irritated by the queen's interference with him, he became exceedingly angry and impertinent. To quiet him the queen promised him he should be made Duke of York. This promise she never meant to keep, and when delay after delay occurred, and the ducal crown was not awarded him, he became very importunate and even threatening. The queen then ordered him off to Ireland to suppress a rebellion there.

He seems to have acquitted himself well in this task, but returned to England suddenly without having first gained the queen's consent. Forcing himself into the queen's presence he once more demanded his dukedom. His manners exasperated the queen, who soundly boxed his ears. He then left her presence and inaugurated a rebellion. This rebellion did not rise above the dignity of a town riot. He was captured and the riot suppressed. He was then tried for high treason, the prosecuting attorney being Lord Bacon, who was literally dragooned into taking this position by the queen. She openly threatened him with death if he did not comply, and so he appeared against his own brother, trusting that the queen would relent at the last moment, and not allow her own son to be executed. He also trusted to his own powers of intercession. But he reckoned without his host. Back of the queen urging her on to extreme steps was her favorite, Robert Cecil, son of Lord Burleigh. This monster in crime and cruelty, having a bitter resentment against Essex, induced the queen to sign his death warrant, and so the Earl was turned over to a couple of blood-thirsty executioners, who first bound him, then with steel levers gouged out both his eyes, after which they led him to the block and beheaded him!

Remorse for the part he had played in the tragedy caused Bacon to record the details of the affair and then to conceal them under the cipher-key in the Shakespearean plays so as to insure the narrative against destruction at the hands of the queen or her successor. It is noticeable that the proceedings of the court where Bacon played his shameful part are carefully omitted from the story as though Bacon could not bear to enter into the details of his own degradation. Yet it contains most severe self-reproach and grief, more deep, more bitter, because of unatoned wrong and unavailing regret. It is one of the most dreadful stories of the cipher revelation.

